# Dual Effects of In Situ Coal Combustion on CaO Pellets for CO_2_ Capture: High-Temperature Sintering and Ash Stabilization

**DOI:** 10.3390/ijms26178535

**Published:** 2025-09-02

**Authors:** Yun Long, Changqing Wang, Ruichang Xu, Lei Liu, Pengxin Zeng, Zijian Zhou, Minghou Xu

**Affiliations:** State Key Laboratory of Coal Combustion, School of Power and Engineering, Huazhong University of Science and Technology, Wuhan 430074, China; m202371276@hust.edu.cn (Y.L.); d202080485@hust.edu.cn (C.W.); m202371275@hust.edu.cn (R.X.); leiliu55@hust.edu.cn (L.L.); d202380528@hust.edu.cn (P.Z.); mhxu@hust.edu.cn (M.X.)

**Keywords:** CaO-based CO_2_ sorbent, in situ coal combustion, deactivation mechanisms

## Abstract

High-temperature CaO-based CO_2_ capture technology, energized by in situ coal combustion, exhibits substantial promise owing to its high energy efficiency, strong compatibility, and maturity. However, sorbent deactivation mechanisms under complex coal combustion conditions, particularly for industrially required pelletized sorbents, are unclear. Pelletized sorbents were co-fired with four representative coals (differing in Na-K, S, and Al-Si content) in this study. Key factors were decoupled, and two competing mechanisms were revealed: (1) High-temperature sintering deactivation: Single co-firing triggers localized overheating (>900 °C), causing severe sintering and pore collapse. This reduces the specific surface area by 29% and pore volume by 50%, occludes meso-/macropores, and leads to a significant drop in initial CO_2_ capture capacity to 0.266–0.297 g/g. Coal types and minor residual surface impurities (<1.7%) are secondary factors. (2) Si-Al ash stabilization: During repeated co-firing (1–9 cycles), Si-Al ash components enrich on sorbents (0.1–7.6%), forming a thermally protective layer. After 20 adsorption–desorption cycles, the CO_2_ capture capacity loss drops from 17.6% to 3.9%, improving cycle stability. These findings clarify these dual mechanisms, providing a theoretical basis for system optimization and highlighting precise control of the combustion temperature field as critical for industrial deployment.

## 1. Introduction

**Figure 1:**
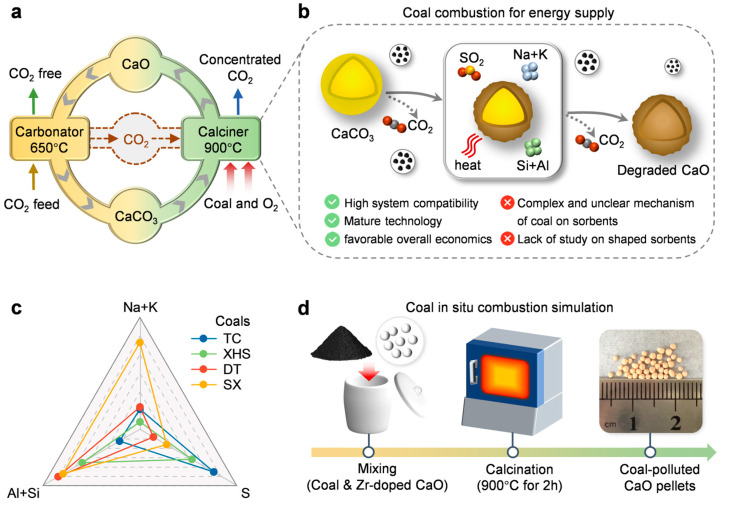
**Coal-integrated calcium looping system and experimental design**. (**a**) Coal-powered calcium looping CO_2_ capture system. (**b**) Complex effects of coal combustion on calcium-based sorbents. (**c**) Inorganic component contents of representative coals. (**d**) Simulation of in situ coal combustion process.

Fossil fuels will maintain dominance in the global energy structure for decades. Their combustion is the primary source of carbon emissions, necessitating urgent development of efficient CO_2_ capture technologies [1,2,3,4]. Calcium looping (CaL) technology utilizes the reversible reaction of calcium-based sorbents, offering a promising path for efficient, low-cost, and environmentally friendly CO_2_ capture [5,6,7,8]. As shown in Figure 1a, this process captures CO_2_ in a carbonator (CaO + CO_2_ → CaCO_3_, 650 °C) and decomposes CaCO_3_ in a calciner (CaCO_3_ → CaO + CO_2_, 800–900 °C) [9,10,11]. This high-temperature requirement creates significant energy demands [12,13]. Using coal oxy-combustion for direct energy supply to the calciner offers three key advantages: stronger system compatibility, superior technological maturity, and favorable overall economics [9,14,15]. While the process incurs an energy penalty, studies indicate that this can be mitigated via thermal integration. For instance, Yang et al. showed that in a 600 MW coal-fired power plant, integrating CaL with waste heat recovery limits the net efficiency loss to within 3.8 percentage points [16]. However, the complex coal combustion environment, including temperature fluctuations, harmful gas emissions, and coal ash deposition, can accelerate the sintering of adsorbents, thereby leading to a decrease in CO_2_ capture performance.

Prior work demonstrates that coal combustion accelerates sorbent deactivation via two pathways: physical thermal sintering and chemical degradation (Figure 1b). Physically, localized overheating and thermal gradients during combustion drive rapid thermal sintering, which collapses the pore structure critical for efficient CO_2_ diffusion and surface reactivity [17,18,19]. Chemically, inorganic components in coal trigger reactions, including liquid-phase sintering (via volatile Na/K forming low-melting eutectics) [20,21], sulfation (SO_2_ reacting to form thermodynamically stable CaSO_4_) [22,23], and aluminosilicate formation (Si/Al oxides reacting with CaO) [24,25,26]. However, the complex interplay of these mechanisms obscures the dominant deactivation pathways under realistic industrial conditions, thereby hindering targeted optimization of sorbents. Existing studies predominantly rely on single coal sources with fixed compositions, which fails to disentangle the relative contributions of distinct pollutants to sorbent degradation, thus impeding the clear identification of key deleterious factors. Moreover, the industrial deployment of CaL necessitates the use of shaped sorbents; however, research on their CO_2_ capture performance and mechanical stability under in situ coal combustion conditions remains scarce [27,28,29].

Four types of coal with distinctly different contents of alkali metals (Na, K), sulfur (S), and aluminum (Al)–silicon (Si) were innovatively employed in this work to disentangle the individual effects of distinct pollutants on sorbent performance, and the dominant pathways governing sorbent deactivation under in situ coal combustion conditions were thereby identified (Figure 1c). The base material used was a pre-optimized, 10% Zr-doped limestone-based pelletized sorbent, which has been demonstrated in previous studies to exhibit enhanced cyclic stability and superior CO_2_ capture capacity [30]. Simulated coal combustion conditions were reproduced in a laboratory muffle furnace environment (Figure 1d). Combined performance testing and microstructure characterization reveal a dual mechanism through which coal influences the sorbent pellets: high-temperature sintering leads to performance degradation, while Al-Si components in the coal ash enhance the CO_2_ capture stability and mechanical strength. These insights provide critical guidance for the targeted design of high-performance calcium-based sorbents and facilitate the practical deployment of CaL for large-scale CO_2_ capture.

## 2. Results

### 2.1. Performance After First Co-Firing

**Figure 2:**
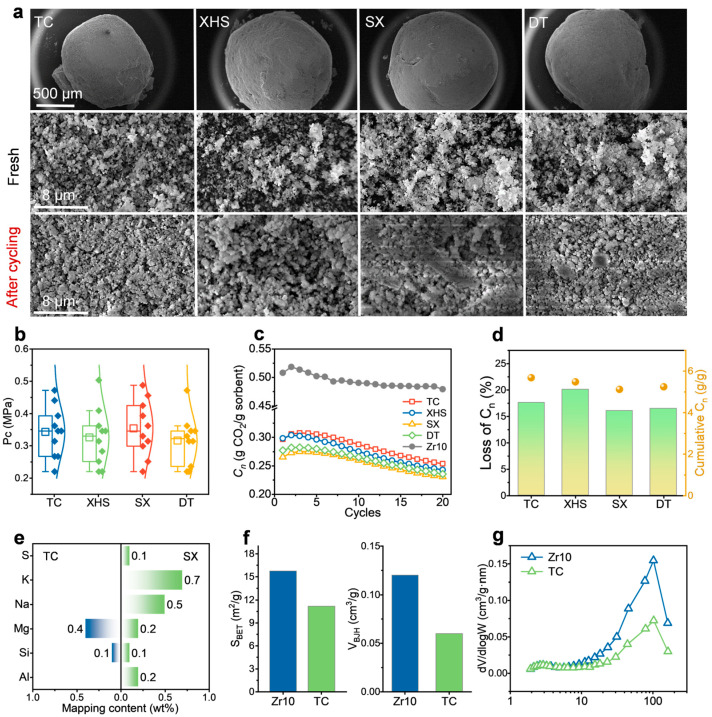
**Single co-firing of coal and sorbent pellets**. (**a**) Morphologies before and after cycling. (**b**) Mechanical strength. (**c**) C_n_ over 20 cycles. (**d**) C_n_ loss (L_n_) and cumulative C_n_ over 20 cycles. (**e**) Inorganic component contents on TC and SX surfaces. (**f**) BET and BJH. (**g**) Pore size distribution.

Coal-contaminated sorbent pellets with a diameter of 0.90 to 1.25 mm were successfully prepared. As shown in Figure 2a, after one cycle of co-firing with coal, the pellets retained an intact spherical shape with partial porous structures remaining on their surface. After 20 carbonation/calcination cycles, the pore structure collapsed, and significant agglomeration and adhesion occurred on the surface. Mechanical properties, a key indicator for engineering applications, were evaluated. Figure 2b shows that the compressive strength (P_c_) follows a normal distribution. The average compressive strength ($\bar{P_{c}}$) values of pellets corresponding to the TC, XHS, SX, and DT coal types are comparable (0.316 to 0.354 MPa), indicating that the coal type has a small effect on mechanical strength. Changes in CO_2_ capture capacity (C_n_) are presented in Figure 2c. Over 20 cycles, the C_n_ of all pellets decreased continuously, consistent with the pore deterioration observed via SEM characterization. The initial C_n_ of coal-blended pellets was significantly lower than that of the coal-free reference sample Zr10. For instance, TC (0.297 g/g), XHS (0.299 g/g), SX (0.266 g/g), and DT (0.277 g/g) showed a ~40% decrease relative to Zr10 (0.508 g/g), indicating that pretreatment via coal co-firing causes significant deterioration in capture performance. Figure 2d shows that the C_n_ loss (L_n_) and cumulative C_n_ over 20 cycles fall within comparable ranges: 16.1% to 17.6% and 5.120 g/g to 5.676 g/g, respectively. Their standard deviations account for 0.102 and 0.046 of the mean values, with minor dispersion, further confirming that the coal type has a limited effect on CO_2_ capture performance.

EDS mapping was used to analyze the surface chemical environment of the pellets. As shown in Figure 2e, the surface environments of TC and SX, which exhibit the largest difference in cumulative C_n_, were compared. TC had residual Si (0.1%) and Mg (0.4%) on its surface, and SX had residual Na-K (1.2%), Al-Si (0.3%), and S (0.1%). Notably, TC (a high-alkali coal) showed minimal residual Na and K, while XHS (a high-sulfur coal) also had little residual S on the surface. This indicates that the coal type is not the dominant factor controlling surface composition; instead, the actual residual depends on the co-firing process. Meanwhile, this trace residual (total content < 1.7%) is significantly lower than that in powder sorbent, attributed to the high resistance to impurity adhesion on pelletized particle surfaces and their strong protection of internal pores. Previous studies attributed performance degradation in powder sorbent primarily to impurities, but this work shows that the extremely low impurity residue on particle surfaces is far from sufficient to explain the ~40% performance attenuation observed.

Further analysis of pore structure evolution was conducted. As shown in Figure 2f, the fresh Zr10 sample had a specific surface area and pore volume of 15.75 m^2^/g and 0.12 m^3^/g, respectively, which are 1.4 and 2.0 times those of the coal-contaminated sample. The pore size distribution in Figure 2g indicates that Zr10 is rich in more mesopores (2 to 50 nm) and macropores (50 to 100 nm), which serve as key channels for CO_2_ adsorption and diffusion [31]. Occlusion of these channels directly leads to capture performance deterioration. Excluding the influence of trace impurities, localized overheating on particle surfaces induced by coal co-firing at up to 900 °C, which results in pore channel closure, is the critical mechanism underlying the decline in CO_2_ capture performance.

### 2.2. Performance After Multiple Co-Firings

**Figure 3:**
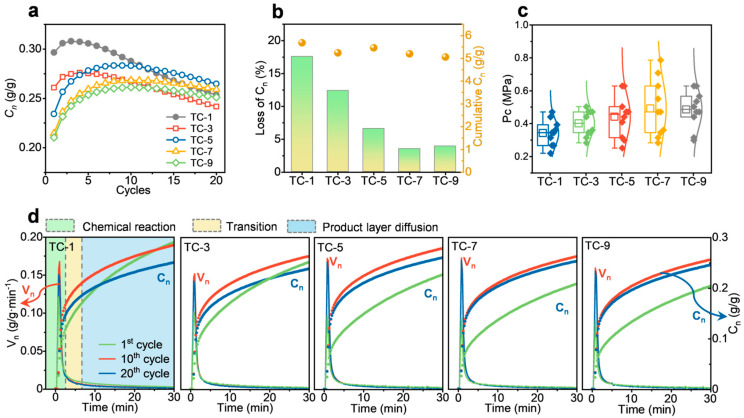
**Multiple co-firing of coal and sorbent pellets**. (**a**) C_n_ over 20 cycles. (**b**) C_n_ loss (L_n_) and cumulative C_n_ over 20 cycles. (**c**) Mechanical strength. (**d**) C_n_ (monotonic curve) and V_n_ (peaked curve) of the 1st, 10th, and 20th cycles.

The TC with the highest cumulative CO_2_ capture capacity was selected for multiple co-firing experiments to simulate the repeated use process of the sorbent. As shown in Figure 3a, the trend of C_n_ over 20 cycles changed significantly with the number of co-firing cycles. First, as the number of co-firing cycles increased from one to three, five, seven, and nine, the C_n_ in the initial cycle gradually decreased from 0.297 to 0.261, 0.234, 0.215, and 0.210 g/g, respectively. Obvious self-activation occurred in subsequent cycles, and the degree of self-activation increased with the number of co-firing cycles. This is because coal combustion exposes the sorbent to high temperatures, promoting the formation of a rigid interconnected hard framework inside the sorbent [32]. Therefore, this framework hindered CO_2_ diffusion in the initial cycles, preventing the CO_2_ capture capacity from reaching the maximum. In subsequent cycles, the formation and decomposition of CaCO_3_ facilitated the growth of an external soft framework, accelerating the carbonation rate and increasing CO_2_ capture capacity, thereby inducing the self-activation of the sorbent [33]. Further analysis was conducted on L_n_ and cumulative C_n_. As shown in Figure 3b, the cumulative C_n_ slightly decreased from 5.676 to 5.053 g/g with the increase in the number of co-firing cycles. L_n_ gradually decreased from 17.6% to 12.4%, 6.6%, and 3.5% as the number of co-firing cycles increased from one to three, five, and seven, with a slight increase to 3.9% at the ninth cycle. This indicates that increasing the number of co-firing cycles slows down the decrease in C_n_, improving cycle stability. Compressive strength was further evaluated, as shown in Figure 3c. With the increase in the number of co-firing cycles from one to three, five, and seven, the average strength rose from 0.343 MPa to 0.401, 0.442, and 0.492 MPa, respectively, before slightly decreasing to 0.486 MPa at nine cycles. This indicates that more co-firing cycles enhance compressive strength.

The dynamic characteristics of C_n_ were further evaluated by analyzing the rate change curve (V_n_). As shown in Figure 3d, V_n_ exhibits a peak-shaped curve that first increases and then decreases. The rate equation theory based on discrete three-dimensional product islands indicates that CO_2_ adsorption by CaO is a typical gas–solid reaction controlled by a combination of chemical reaction and product layer diffusion, involving three stages: chemical reaction, transition, and product layer diffusion [34,35]. As the number of cycles increased from 1 to 10 and then to 20, the peaks of V_n_ for all samples first increased and then decreased, consistent with the self-activation phenomenon of C_n_ mentioned above. With the increase in the number of co-firing cycles, the dynamic curves of V_n_ and C_n_ over time for the 10th and 20th cycles increasingly overlapped, further indicating enhanced cycle stability.

**Figure 4:**
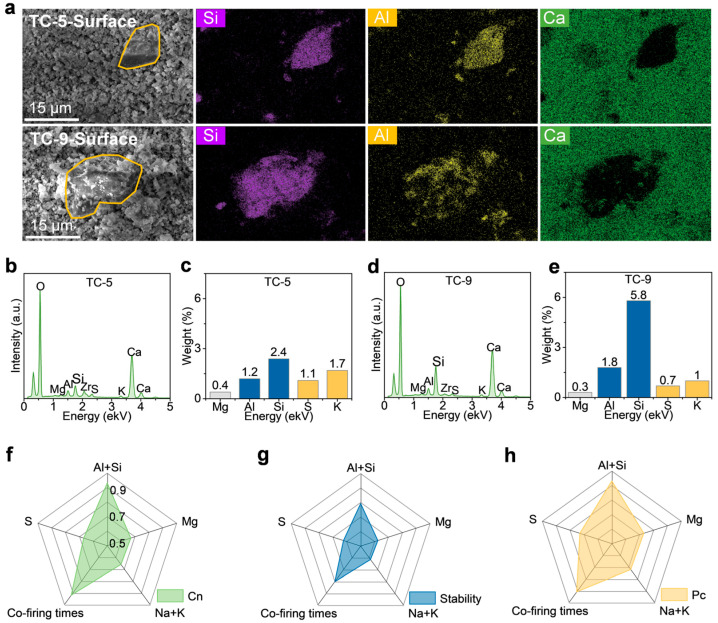
The 5th and 9th co-firing cycles. (**a**) SEM and EDS mapping for the 5th co-firing cycle. (**b**) Energy spectrum and (**c**) elemental contents for the 9th co-firing cycle. (**d**) Energy spectrum and (**e**) elemental contents. Gray relational analysis. (**f**) C_n_ vs. multiple factors. (**g**) Cyclic stability vs. multiple factors. (**h**) Mechanical strength vs. multiple factors.

EDS mapping was used to analyze the effect of co-firing cycles on the surface chemical environment of sorbent pellets. As shown in Figure 4a, significant changes in the surface chemical environment occurred after five co-firing cycles, particularly the surface agglomeration of Al- and Si-based oxides. Figure 4b–e show the element energy spectra and content comparison. The energy spectrum intensity of Al and Si elements increased significantly with the number of co-firing cycles, with their contents increasing from 0.1% in the first co-firing to 3.6% in the fifth and further to 7.6% in the ninth. The contents of alkali metals, S, and Mg remained below 1.7%. This is attributed to the fact that silico-aluminum oxides account for 52.68–90.82% of coal ash. Notably, unlike powdered sorbents, silico-aluminum oxides only adhere to the surface of sorbent pellets, without consuming active CaO components.

Furthermore, gray correlation analysis was performed to investigate the relationships between four influencing factors (number of co-firing cycles, representing high temperature; Al and Si; Na and K; and S) and three performance metrics (C_n_, cycle stability, and P_c_). As shown in Figure 4f–h, the number of co-firing cycles and Al-Si content exhibited strong correlations with the performance metrics. The negative correlation degrees of co-firing cycles and Al/Si content with cumulative C_n_ were 0.91 and 0.93; their positive correlation degrees with cycle stability were 0.80 and 0.79; and their positive correlation degrees with P_c_ were 0.91 and 0.93, respectively. Therefore, the content of Al and Si increases with the increase in the number of co-firing cycles, leading to a slight decrease in cumulative C_n_, a significant improvement in cycle stability, and a certain enhancement in mechanical properties. It is worth noting that the main reason for the improved cycle stability is the agglomeration of high-temperature-resistant Si-Al oxides on the surface of sorbent pellets, which plays a role in retarding sintering.

## 3. Discussion

**Figure 5:**
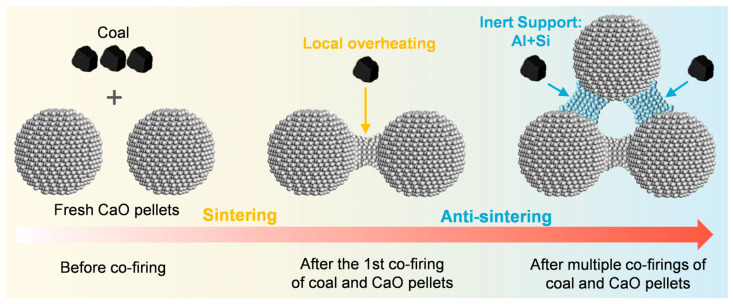
Dual competing mechanisms: high-temperature sintering deactivation and Si-Al ash stabilization.

Single co-firing significantly degrades the CO_2_ capture performance of sorbent pellets. The key influencing factors are the sintering and pore structure collapse of sorbent pellets induced by localized overheating during coal combustion, with a minor correlation with changes in the surface chemical environment or coal types (Figure 5). To meet the requirement of repeated use in practical applications, multiple cycles of co-firing were conducted. It was found that the content of Al and Si on the surface gradually increases, forming a high-temperature-resistant protective layer and significantly improving cycle stability (Figure 5). Considering the competing effects of temperature-induced degradation and stability enhancement by aluminosilicate oxides, future research should prioritize identifying a critical temperature window. This window can balance sintering suppression and stability promotion, which will be achieved through fixed-bed breakthrough experiments. Such studies will provide a theoretical basis for the long-term operation of sorbents. Notably, while the high temperatures of coal combustion degrade sorbent performance, activation strategies such as steam treatment can effectively revitalize these materials [36,37].

## 4. Materials and Methods

### 4.1. Coal Selection

Four types of coal were selected as research objects: Tianchi coal, Xiheishan coal, Datong coal, and Shanxi coal. The coal samples were pulverized via grinding and sieved to collect coal pellets smaller than 100 μm. After drying at 60 °C, the samples for combustion were obtained. The proximate and ultimate analysis results of the four coals are presented in Table 1. Ultimate analysis results show that Shanxi coal has the highest S content, reaching 4.65 wt%, while the S contents of the other coal samples are all below 1.20 wt%. Coal ash was obtained using plasma low-temperature ashing technology at temperatures below 200 °C with the aim of minimizing the loss of volatile alkali metals (Na and K) in the coal. The ash composition was analyzed via X-ray fluorescence spectroscopy (XRF, Bruker, Bremen, Germany), and the results are given in Table 2. Tianchi coal and Xiheishan coal have relatively high alkali contents (calculated as Na_2_O and K_2_O), exceeding 5.39 wt%. Datong coal has the lowest alkali content at 1.40 wt%, while Shanxi coal has a slightly higher alkali content of 2.76 wt%. Meanwhile, the content of silico-aluminum oxides is relatively low in Tianchi coal (52.48 wt%), whereas that in all the other coals exceeds 75.89 wt%.

### 4.2. In Situ Coal Combustion Simulation

First, pulverized coal was mechanically mixed with pelletized, Zr-loaded, limestone-derived sorbents (from previous work) at a specific ratio. The mixed samples were then placed in a muffle furnace and calcined at 900 °C for 2 h in an air atmosphere to simulate in situ coal calcination conditions. As shown in Figure 1d, after calcination, pelletized, limestone-derived sorbents with coal ash adhered to their surfaces were obtained, with a uniform particle size distribution of 0.9–1.25 mm. Sorbent pellets contaminated by Tianchi coal, Xiheishan coal, Datong coal, and Shanxi coal are designated as TC, XHS, DT, and SX, respectively. Multiple co-firing cycles involved re-adding coal followed by repeated calcination. Sorbent pellets after the third, fifth, seventh, and ninth calcination cycles were selected to investigate their CO_2_ capture performance. Under theoretical conditions, all heat released from complete coal combustion is used for the thermal decomposition of CaCO_3_. The formula for calculating the required coal mass is as follows:

(1) $$\frac{m_{\mathrm{coal}}}{m_{{\mathrm{CaCO}}_{3}}}=\frac{∆H}{M_{{\mathrm{CaCO}}_{3}}}\times \frac{1000}{Q}$$

where $m_{\mathrm{coal}}$ and $m_{{\mathrm{CaCO}}_{3}}$ are the masses of coal and the pelletized sorbent (converted to calcium carbonate), respectively, kg; ∆H is the heat absorption for the decomposition of 1 mol of CaCO_3_, 178 kJ/mol; $M_{{\mathrm{CaCO}}_{3}}$ is the molar mass of CaCO_3_, 100 g/mol; and Q is the low calorific value of coal, kJ/kg. The calculated mass ratios of added Tianchi coal, Xiheishan coal, Dadong coal, and Shanxi coal were 8.4 wt%, 10.4 wt%, 7.5 wt%, and 9.2 wt%, respectively.

### 4.3. CO_2_ Capture Capacity Testing

CO_2_ capture performance tests were conducted on a simultaneous thermal analyzer (STA 449 F5 Jupiter, NETZSCH, Selb, Germany), which enables simultaneous acquisition of mass and heat flow data for materials in a single test. Each sample underwent 20 cycles of carbonation (650 °C, 15 vol% CO_2_, 30 min) and calcination (850 °C, 100 vol% N_2_, 5 min), with N_2_ as the balance gas. The heating and cooling rates were 20 °C/min. Performance evaluation metrics included the CO_2_ capture capacity ($C_{n}$, g CO_2_/g sorbent), CO_2_ capture capacity loss ($L_{n}$, %), and CO_2_ adsorption rate ($V_{n}$, g CO_2_ g^−1^ sorbent min^−1^). The specific calculation formulas are as follows:

(2) $$C_{n}=\frac{m_{\mathrm{car},n}-m_{\mathrm{cal},n}}{m_{\mathrm{cal},n}}\;\times \;100\%\;$$

(3) $$V_{n}=\frac{dC_{n(t)}}{\mathrm{dt}}$$

(4) $$L_{n}=\frac{C_{\max}-C_{n}}{C_{\max}}\times 100\%$$

where $m_{\mathrm{car},n}$ and $m_{\mathrm{cal},n}$ are the masses of the sample during the nth carbonation and calcination cycles, respectively, g; t is the carbonation time, min; $C_{n(t)}$ is the time-dependent $C_{n}$, g/g; and $L_{n}$ is the percentage loss of the nth cycle $C_{n}$ relative to the maximum $C_{n}$ ($C_{\max}$), %.

### 4.4. Mechanical Property Testing

Mechanical properties were tested using a precision pressure testing machine (DL5 type intelligent particle strength tester, Dalian chemical engineering research and design institute, China), with the metric being the maximum compressive strength at particle breakage ($P_{c,i}$, MPa). $P_{c,i}$ is defined as the ratio of the maximum breaking force ($F_{\max}$) to the average cross-sectional area of the particle. Ten sorbent pellets with a particle size of 0.9–1.25 mm were subjected to breakage resistance tests. The average compressive strength ($\bar{P_{c}}$) is calculated as follows:

(5) $$P_{c,i}=\frac{F_{\max}}{\pi r^{2}}$$

(6) $$\bar{P_{c}}=\sum_{i=1}^{n}P_{c,i}/n$$

where $F_{\max}$ is the maximum force required to crush a single particle, N, and r is the average radius of the CaO pellets, mm.

### 4.5. Gray Relational Analysis

First, dimensionless processing is performed on the data sequence of each factor. After processing, dimensionless data (such as $x_{C_{n}}$ and $x_{factors}$ in the formula) are obtained, which normalizes the values of different factors in proportion to facilitate subsequent comparison.

(7) $${∆}_{C_{n}-factors}(t)=\left|x_{C_{n}}\left(t\right)-x_{factors}\left(t\right)\right|,t=1,2,3$$

Next, the deviation sequence is calculated to quantify the differences between the reference sequences and the factor sequences. The reference sequences correspond to the target performance metrics of interest, namely, three items: C_n_, cycling stability, and P_c_. The factor sequences are the comparison sequences, namely, five items: co-firing times, Na-K, Al-Si, Mg, and S. Then, the global maximum deviation ${∆}_{max}$ and global minimum deviation ${∆}_{max}$ are identified.

(8) $${∆}_{max}={max}_{factors}({max}_{t}\left|x_{C_{n}}\left(t\right)-x_{factors}\left(t\right)\right|)$$

(9) $${∆}_{min}={min}_{factors}({min}_{t}\left|x_{C_{n}}\left(t\right)-x_{factors}\left(t\right)\right|)$$

The gray relational coefficients are then computed using the Formula (9). ρ is the resolution coefficient, typically set to 0.5.

(10) $$\xi_{C_{n}-factors}(t)=\frac{{∆}_{min}+{\rho ∆}_{max}}{{∆}_{C_{n}-factors}(t)+{\rho ∆}_{max}}$$

Finally, the gray relational degree is computed to represent the overall correlation between a factor sequence and the reference sequences. The average of the gray relational coefficients across all points t is taken.

(11) $$\gamma_{C_{n}-factors}=1/3\sum_{t=1}^{3}\xi_{C_{n}-factors}\left(t\right)$$

### 4.6. Characterization

Semi-quantitative analysis was conducted with X-ray fluorescence (XRF, Bruker, Germany). The surface microtopography of samples before and after cycling was characterized using a Phenom Star benchtop scanning electron microscope (SEM, Phenom Star benchtop, Eindhoven, The Netherlands) operated at a 3 kV accelerating voltage. The elemental composition and distribution within samples were ascertained via energy-dispersive X-ray spectroscopy (EDX). The specific surface area and pore volumes were determined using a N_2_ adsorption-desorption analyzer (Micromeritics ASAP2020, Norcross, GA, USA), applying the Brunauer-Emmett-Teller (BET) method for surface area and the Barrett-Joyner-Halenda (BJH) method for pore volume calculations.

## 5. Conclusions

In this work, four coals representing three key compositional groups (alkali metals, sulfur, and Al/Si) were selected to explore the mechanisms governing the effects of single and multiple co-firing cycles between coal and pelletized sorbents. Single co-firing triggered particle sintering and pore collapse due to localized combustion overheating (900 °C), leading to a 29% reduction in the specific surface area and a 50% decrease in pore volume. Closure of mesopore/macropore channels caused a notable drop in initial CO_2_ capture capacity to 0.266–0.299 g/g. Importantly, neither trace surface impurity residues (<1.7%) nor differences in the coal type were primary causes of performance degradation. During multiple co-firing cycles, Al-Si oxides gradually accumulated on sorbent surfaces (rising from 0.1% to 7.6% after one to nine cycles), forming a high-temperature-resistant protective layer. This led to significant improvements in cyclic stability, with the CO_2_ capture capacity loss decreasing from 17.6% to 3.9% over 20 cycles. Thus, in situ coal combustion exerts dual effects on adsorbent particles: degradation induced by high-temperature sintering and cyclic stability enhanced by Si-Al components in the ash.

## Figures and Tables

**Table 1:** Proximate and ultimate analyses of various coal samples (air-dried basis, wt%).

	Proximate Analysis				Ultimate Analysis					Low Calorific Value Q (kJ/kg)
	Mad	Vad	Aad	FCad	Cad	Had	Oad	Nad	Sad	
Tianchi coal	13.62	33.92	12.22	40.24	55.44	4.37	38.57	0.55	1.07	21,098
Xiheishan coal	13.15	26.90	25.53	34.42	44.22	3.88	50.96	0.54	0.40	17,121
Datong coal	2.10	38.10	27.20	32.60	75.00	4.60	17.90	1.30	1.20	23,862
Shanxi coal	3.14	16.57	11.95	68.34	53.87	3.77	36.84	0.87	4.65	19,327

Mad stands for moisture content; Vad is volatile matter; Aad refers to ash content; FCad is fixed carbon; Cad denotes carbon content; Had is hydrogen content; Oad represents oxygen content (note: oxygen content is calculated according to the difference); Nad is nitrogen content; and Sad refers to sulfur content.

**Table 2:** XRF analysis results of low-temperature ash compositions of various coal samples (wt%).

	Na_2_O	K_2_O	MgO	Al_2_O_3_	SiO_2_	P_2_O_5_	Cl	CaO	Fe_2_O_3_
Tianchi coal	6.90	0.75	2.75	13.82	38.86	1.18	1.16	16.83	17.74
Xiheishan coal	3.74	1.65	3.91	18.54	57.35	1.15	0.74	4.90	8.03
Datong coal	0.95	0.45	1.04	35.45	55.37	0.44	0.00	2.70	3.60
Shanxi coal	1.73	1.03	1.51	36.09	51.65	0.47	0.00	3.89	3.62

## Data Availability

The data presented in this study are available on request from the corresponding author.
